# Investigating determinants of out-of-pocket spending and strategies for coping with payments for healthcare in southeast Nigeria

**DOI:** 10.1186/1472-6963-10-67

**Published:** 2010-03-17

**Authors:** Obinna E Onwujekwe, Benjamin SC Uzochukwu, Eric N Obikeze, Ijeoma Okoronkwo, Ogbonnia G Ochonma, Chima A Onoka, Grace Madubuko, Chijioke Okoli

**Affiliations:** 1 Department of Health Administration and Management, College of Medicine, University of Nigeria, Enugu, Nigeria; 2 Health Policy Research Group, Department of Pharmacology and Therapeutics, College of Medicine, University of Nigeria, Enugu, Nigeria; 3 Department of Community Medicine, College of Medicine, University of Nigeria, Enugu, Nigeria; 4 Department of Nursing, College of Medicine, University of Nigeria, Enugu, Nigeria

## Abstract

**Background:**

Out-of-pocket spending (OOPS) is the major payment strategy for healthcare in Nigeria. Hence, the paper assessed the determinants socio-economic status (SES) of OOPS and strategies for coping with payments for healthcare in urban, semi-urban and rural areas of southeast Nigeria. This paper provides information that would be required to improve financial accessibility and equity in financing within the public health care system.

**Methods:**

The study areas were three rural and three urban areas from Ebonyi and Enugu states in South-east Nigeria. Cross-sectional survey using interviewer-administered questionnaires to randomly selected householders was the study tool. A socio-economic status (SES) index that was developed using principal components analysis was used to examine levels of inequity in OOPS and regression analysis was used to examine the determinants of use of OOPS.

**Results:**

All the SES groups equally sought healthcare when they needed to. However, the poorest households were most likely to use low level and informal providers such as traditional healers, whilst the least poor households were more likely to use the services of higher level and formal providers such as health centres and hospitals. The better-off SES more than worse-off SES groups used OOPS to pay for healthcare. The use of own money was the commonest payment-coping mechanism in the three communities. The sales of movable household assets or land were not commonly used as payment-coping mechanisms. Decreasing SES was associated with increased sale of household assets to cope with payment for healthcare in one of the communities. Fee exemptions and subsidies were almost non-existent as coping mechanisms in this study

**Conclusions:**

There is the need to reduce OOPS and channel and improve equity in healthcare financing by designing and implementing payment strategies that will assure financial risk protection of the poor such pre-payment mechanisms with government paying for the poor.

## Background

Out-of-pocket spending (OOPS) is the major payment strategy for healthcare in Nigeria. The real challenge of health care financing in Nigeria as in many countries in sub-Saharan Africa (SSA) lies not primarily in the acute scarcity of resources, but in the absence of intermediation and insurance mechanisms to manage risk, and inefficient resource allocation and purchasing practices [1]. User fees fall within the broader concept of "cost-sharing", a practice whereby beneficiaries contribute towards the cost of a public service and they are defined as payment of out-of-pocket charges at the time of use of services [2].

OOPS for healthcare increased with the introduction of user fees in the health sector and like most African countries, Nigeria introduced user fees as a mode of financing government health services within the framework of the Bamako Initiative revolving drug funds [3]. It is however noted that user fees and revolving drug funds are interlinked. The introduction of user fees was arguably in response to the severe problems in financing health services in Nigeria, like in most of sub-Saharan Africa. Government health budgets declined in real terms in response to macroeconomic problems at the time while demand for health services increased, partly because of population growth and successful social mobilization. Currently, user fees apply to government owned healthcare services in Nigeria with the major aim being to generate more funds for the health sector, so as to improve the quality of services [4]. In the private sector, patients are also charged fees which they mostly pay out-of-pocket.

However, public expenditures in Nigeria account for just 20-30% of total health expenditures (THE), whilst private expenditures accounts for 70-80% of THE and the dominant private expenditure is OOPS, which accounts for more than 90% of private health expenditures [1,5]. The recently introduced national health insurance scheme (NHIS) is not expected to change the picture in the near future since it presently covers a minority comprising only federal government civil servants. The NHIS plans to initiate community-based health insurance schemes to cover people employed in the informal sector in some pilot communities in Nigeria soon. The prevailing excessive private share of expenditures in Nigeria is all the more alarming as most of it takes place via non-pooled OOPS, which has been noted as the most regressive form of payment [6]. The composition of THE in Nigeria shows that private expenditures accounted for 66.5% of THE in 2000 and 74.4% in 2002 [1]. The household OOPS as a proportion of private health expenditure, has been more than 90% in all years (Table 1). The Nigerian 2003-2005 National Health Accounts show that private health expenditure still constitutes more than 70% of THE [5]. This can lead to high incidence of catastrophic expenditures [7]. OOPS is about US$ 22.5 per capita, which accounts for 9% of total household expenditures and half of those who could not access care did not so because of its costs [8].

**Table 1:** Private Health Expenditure in Nigeria, 1998-2005^1, 5^

Private Health Expenditures	1998	1999	2000	2001	2002	2003	2004	2005
Private sector expenditure on health as % of Total Expenditure on health	73.9	70.9	66.5	68.6	74.4	81.3	73.6	74.0

Private households' OOPs as % of private sector expenditure on health	95.0	94.8	92.7	91.4	90.4	91.0	89.3	90.9

In most developing countries, OOPS are regressive while social assistance and fee exemptions are either non-existent or where present, are not well targeted at those most in need [9-11]. The absence of exemption mechanisms and pre-paid instruments is largely responsible for impoverishing health expenditures [6]. With 70% of the population in Nigeria living below the $1-a-day [12], the excessive reliance on OOPS curbs health care consumption, exacerbates the already inequitable access to quality care, and exposes households to the financial risk of expensive illness at a time when there are both affordable and effective health financing instruments to address such problems in low income settings [6].

Some analysts and donors have argued that introducing national user fee systems would address inefficiencies and inequities in the health system [13-16]. It is recognized that a user fee policy should include provisions for exemption of the poor. However, implementation of exemption systems is fraught with problems, including identifying the eligible poor and administrative incapacity [17]. Distinguishing the poor from the non-poor poses practical problems that can render the application of waivers and exemption system ineffective. Hence, a key factor underlying the inequitable impact of fees was the failure of nearly all governments to design or implement targeted exemption mechanisms for the poor [18,19].

There is paucity of information on how OOPS and other financing mechanisms lead to or have differential effect on various socio-economic classes in healthcare seeking, access to care and utilization of services in Nigeria. The federal government and some state governments have abolished user fees for the treatment of some diseases such as malaria for the under-fives and pregnant women in the public sector. However, user fees are still in place for the general population and even for some services for the under fives and pregnant women. In Uganda, after user fees were abolished in 2001, the observed increase in utilization of health services was more among the poor than in other socio-economic categories [11]. Some developing countries such as Viet Nam, Guatemala, India, Mexico, Nepal and South Africa adopted other pro-poor payment systems such as waivers and exemptions in response to the negative impact of user fees [10].

The objective of this study was to assess the incidence and determinants of OOPS with emphasis on the SES differences in occurrence and coping mechanisms for OOPS and other household payment mechanisms. Thus, this paper provides information that would be required for improved financial accessibility and equity in financing within the public health care system, especially within primary healthcare (PHC) which is the cornerstone of the Nigerian health care system [4]. Generating new knowledge about the determinants and effects of OOPS in access to and utilization of PHC services, as well as mechanisms to protect the poor from the adverse effects of OOPS is both a national and international priority.

## Methods

### Study area

The study areas were Ebonyi and Enugu states which lie within the South-east health care zone comprising of 5 states. The study states combined reflect the situation in Nigeria. In each state, an urban, a semi-urban and a rural community were selected. Hence, six communities were purposively selected in the two states (so as to be representative of different geographic settings). These were the two state capitals (Enugu and Abakiliki), two local government area (LGA) headquarters (Udi in Enugu state and Ezilo in Ebonyi state) and two rural communities (Eke-na-ene in Enugu state and Nkalagu in Ebonyi state). Nigeria has an estimated population of 140 million people. Enugu has a population of about 800,000 people, while Abakiliki has about 500,000 residents. Each LGA headquarters has a minimum population of 40,000 people, while each rural community has a minimum population of 10,000 people. While trading and civil service work are the major sources of income in Enugu and Abakiliki, subsistence farming and petty trading are the major sources of livelihood in the other four communities.

Each study site has at least one primary health care centre. In addition, the two state capitals are served by a teaching hospital while the two Local Government headquarters are served by a public general Hospital. There are also a number of private hospitals/clinics, patent medicine dealers, and a wide range of private healthcare providers (including traditional medicine practitioners) in each study community.

### Household survey

The sample size was determined using the formula for sample size for a definite population, considering 0.25 as the proportion of the population positive for the health conditions that will require payments, power of 80%, confidence interval of 95% and 0.05 as the absolute sampling error that can be tolerated. Hence, 300 households represented an adequate sample size per study area. However, in order to take care of refusals, the primary healthcare (PHC) house numbering system was used as the sampling frame to select 380 households from each urban site and 330 from each rural site using simple random sampling. Using a pre-tested questionnaire (see additional file 1), data was collected from the primary woman household care-giver or in her absence, her spouse. The questionnaires were administered by trained interviewers who were residents of the various communities.

The questionnaire was pre-tested amongst 50 residents of a peri-urban community near Enugu and the results were used to improve some of the language used in the questionnaire, some questions, the mode of questioning and the coding of some responses. We arrived at the decision to use some specific household assets and weekly food expenditure to determine socio-economic status after discussions with many key informants from the communities.

The questionnaire explored the demographic and socio-economic characteristics of respondents and their households. The questionnaire was also used to examine the healthcare seeking practices. Healthcare seeking was measured as number of cases in household in a one month period that sought healthcare from different providers. It did not include self-treatment. The expenditures (transportation and actual treatment) to pay for primary healthcare services for the diseases or health conditions were determined using the one-month recall period. Some questions were used to determine the payment strategies that people used to pay for healthcare and how they coped with the payments. The payment mechanisms explored were direct payments with reimbursement by employers (reimbursement), out-of-pocket spending (OOPS), National Health Insurance Scheme (NHIS), community-based health insurance (CBHI), private voluntary health insurance (PVHI) payment in kind and payment by installment.

The approval for the study was obtained from the University of Nigeria Research Ethics Committee. Verbal informed consent was obtained from all the respondents, who were all given the option of not participating in the study if they so wished

### Data analysis

Principal components analysis (PCA) in STATA software package [20] was used to create a continuous socio-economic status (SES) index [21,22] using information from the households' asset holdings together with the per capita weekly cost of food. The first principal component of the PCA was used to derive weights for the SES index [23]. The assets were ownership of motorcar, motorcycle, radio, refrigerator, television set and bicycle. In addition the monthly value of food was also included. The SES index was divided into SES quartiles. Income was not used in defining SES because people in Nigeria do not provide reliable information about their income. Testing of means was used to divide some key variables into the SES quartiles and Kruskal-Wallis statistics was used to compute chi-square for trends, in order to determine whether the means of the quartiles were statistically significantly different.

The measures of inequity were the ratio of the mean of the poorest SES group (1^st ^quartile) over that of the least poor SES group (4^th ^quartile) (top/bottom quartile ratio) and concentration index [24-26]. The top/bottom (Q1/Q4) quartile ratio shows the level of gap that has to be bridged in order to ensure equity and improve the condition of the poorest households and a score of 1 signifies perfect equity [22]. The concentration index varies from -1 and +1 and a negative sign shows that the variable of interest is higher among the poorest and if positive, it means that it is more among the richest (or least poor).

Logistic regression analysis was used to examine the multivariate relationship of OOPS with key explanatory variables. The dependent variable was whether or not someone paid through OOPS. The explanatory variables were the weight that was used to derive the SES index, households' socio-demographic characteristics and costs of transportation and cost of treatment itself. There were no prior hypothetical expectations about the relationship of the dependent and the explanatory variables, because the analysis was more about hypotheses generation rather than hypotheses testing.

## Results

### Characteristics of the respondents and their households

The number of complete questionnaires available for data analysis were 370, 366, 376, 298, 352 and 300 in Abakiliki, Ezilo, Nkalagu, Eke-na-ene, Enugu and Udi respectively (Table 2). The low number of respondents in Eke-na-ene and Nkalagu (rural areas) was due to the low population there. The reduced number of questionnaires in the six communities when compared with the sample selected was because of some few refusals to be interviewed and refusal to answer some of the questions.

**Table 2:** Respondents' and households' Socio-economic and demographic characteristics

	Abakaliki (urban)		Ezilo (semi-urban)		Nkalagu (rural)		Eke-na-ene (rural)		Enugu (urban)		Udi (semi-urban)	
	**(N = 370)**		**(N = 366)**		**(N = 376)**		**(N = 300)**		**(N = 352)**		**(N = 300)**	

Household heads: n %	163	43.9	341	93.2	104	27.7	199	66.3	118	33.5	236	78.7

No of household residents: Mean (SD)	4.8 (2.5)		6.4 (3.4)		6.8 (4.2)		5.9 (2.8)		4.4(2.0)		5.2(2.54)	

Age of respondent: Mean (SD)	36.6 (11.2)		42.6 (11.7)		42.1 (13.6)		54.8 (14.1)		40.46(13.34)		54.4(13.5)	

Sex (Males)	163	43.9	334	91.3	91	24.2	188	62.7	95 (27.0)		164 (54.7)	

Years of education: Mean (SD)	8.6 (4.7)		6.0 (5.1)		3.3 (5.0)		6.0 (4.9)		11.5 (4.7)		4.5(4.4)	

Whether married: n %	356	96.0	351	95.9	366	97.3	290	96.7	331	94.0	293 (97.7)	

Weekly food cost: Mean (SD)	$15.75 ($10.61)		$17.21 ($18.0)		$26.14 ($28.09)		$10.07 ($16.35)		$16.98 ($11.15)		$8.61 ($9.25)	

Radio: n %	347	93.5	323	88.3	346	92.0	276	92.0	346	98.3	239	79.7

Fridge: n %	176	47.4	26	7.1	30	8.0	96	32.0	309	87.8	48	16.0

TV: n %	296	79.8	96	26.2	60	16.0	177	59.0	325	92.3	95	31.7

Bicycle: n %	84	22.6	206	56.3	260	69.1	46	15.3	6	1.7	37	12.3

Motorcycle: n %	169	45.6	58	15.8	47	12.5	20	6.7	41	11.6	17	5.7

Motorcar: n %	50	13.5	5	1.4	23	6.1	60	20.0	106	30.1	14	4.7

As shown in Table 2, the respondents were mostly heads of households in the rural areas (with the exception of Nkalagu), while representatives of households were the majority of the respondents in the two urban areas (Abakiliki and Enugu). Also, majority of the respondents were males in the rural areas (with the exception of Nkalagu), while the converse was true in the two urban areas. The respondents from the urban areas were expectedly more educated than those from the rural areas. The average years of formal schooling was 11.5 years in Enugu and 8.6 years in Abakiliki while it was 4.5 years, 6.0 years, 3.3 years and 6.0 years in Udi, Eke-na-ene, Ezilo and Nkalagu respectively. Most of the respondents in the six communities were middle-aged. The average number of household residents (average household size) ranged from 4.4 in Enugu to 6.8 in Nkalagu. The average weekly household cost of food ranged from as low as $8.61 in Udi to as high as $26.14 in Nkalagu. The average weekly cost of food per household member was $3.6, $3.0, $4.2, $2.0, 3.5 and $1.2 in Abakiliki, Ezilo, Nkalagu, Eke-na-ene, Enugu and Udi respectively. The higher amounts in some rural areas were due to the high costs of home produced and consumed food items there. The households in the urban areas had better valuable asset holdings than their rural counterparts and were more likely to own a television set, refrigerator and motorcar. The observed socio-economic and demographic characteristics of the study sample reasonably reflect that of the of the study population.

### Health Seeking

The study also shows how the respondent sought for health care (Table 3) in the one-month recall period. The very poor group (28%) sought health care most while the poorest group (22%) were least in health seeking in Abakaliki. In Obinagu, the poorest group (30%) sought for health care most while the remaining percentages were distributed almost equally among the other SES groups in the community. Health care was sought most by those in quartile 3 in Ezilo (28%), Nkalagu (26%), Eke-na-ene (28%) and Enugu (26%). However, concentration indices showed that the variables of interest were in favour of the worse off in Abakaliki and Obinagu, and in favour of the better off in the rest of the study communities. The poorest households were most likely to use low level and informal providers, such as traditional healers, whilst the least poor households were more likely to use the services of high level providers such as health centres and hospitals.

**Table 3:** General Issues about health seeking

	Abakaliki (urban) (%)	Ezilo (semi-urban) (%)	Nkalagu (rural) (%)	Eke-na-ene (rural) (%)	Enugu (urban) (%)	Udi (semi-urban) (%)
Sought healthcare						
Q1 (most poor)	49 (22)	57 (24)	76 (23)	49 (24)	78 (24)	48 (30)
Q2 (very poor)	61 (28)	52 (22)	83 (25)	48 (24)	82 (25)	38 (24)
Q3 (poor)	49 (23)	67 (28)	84 (26)	57 (28)	85 (26)	37 (23)
Q4 (least poor)	59 (27)	63 (26)	85 (26)	48 (24)	80 (25)	36 (23)
Chi-square	5.12	6.05	4.78	3.90	4.77	3.03
p-value	0.16	0.11	0.19	0.27	0.19	0.39
Q1/Q4	0.83	0.90	0.89	1.02	0.98	1.33
Concentration index	-0.24	0.03	0.02	0.01	0.01	-0.04

### Expenditures on healthcare seeking

Higher costs of treatment were incurred in the two urban areas (Abakiliki and Enugu) and the overall mean monthly cost of treatment per respondent ranged from 440.7 Naira (US$4.01) in Udi to 1477.0 Naira (US$13.43) in Enugu. Conversely, the average monthly costs of transportation per respondent were higher in the rural areas and the people paid an average of 35.3 Naira (US$0.32) in Eke-na-ene to 162.3 Naira (US$1.48) in Ezilo. The total treatment costs for other household members mirrored that of the respondents, although the highest costs were incurred in Ezilo and Nkalagu.

### Payment strategies

OOPS was by far the commonest type of payment mechanism that was used by respondents to pay for their healthcare in the six communities (Table 4). This was followed distantly by installment payment, with the exception of Abakiliki where reimbursement was the second most common payment mechanism. The other payment mechanisms were rarely used, though it was interesting to find that a few people claimed to have utilized health insurance, which is still new in Nigeria. The urbanites significantly used more of OOP payment strategy, while the rural dwellers significantly used more of reimbursement, installment payment and in-kind payment mechanisms. As was the case for respondents' payment mechanism for healthcare, OOPS was used for the payment of healthcare for other household members (Table 4). It was again followed in frequency of use by payment by installment.

**Table 4:** Payment mechanisms that were used to pay for healthcare for people that consumed healthcare services

Payment mechanism for the respondents												
	**Abakaliki (urban)**		**Ezilo (semi-urban)**		**Nkalagu (rural)**		**Eke-na-ene (rural)**		**Enugu (urban)**		**Udi (semi-urban)**	
	**N = 229**		**N = 244**		**N = 333**		**N = 209**		**N = 321**		**N = 174**	
	**n**	**(%)**	**n**	**(%)**	**n**	**(%)**	**n**	**(%)**	**n**	**(%)**	**n**	**(%)**

OOPs	210	91.7	205	56.0	273	2.0	184	88.0	311	96.9	119	68.4

Reimbursement	6	2.6	7	4.6	19	5.7	6	2.9	2	0.6	15	8.6

Health insurance	0	0	3	0.8	1	0.3	1	0.5	3	0.9	7	4.0

Installment payment	0	0	15	4.1	17	5.1	18	8.6	24	7.5	30	17.2

In-kind	0	0	2	0.5	4	1.2	2	1.0	2	0.6	1	0.6

Others	13	5.7	9	2.4	19	5.7	9	4.4	9	2.7	2	1.2

**Payment mechanism for other household members**												

	**N = 268**		**N = 253**		**N = 349**		**N = 192**		**N = 326**		**N = 148**	
	**n**	**(%)**	**n**	**(%)**	**n**	**(%)**	**n**	**(%)**	**n**	**(%)**	**n**	**(%)**

OOPS	265	98.9	204	80.6	308	88.3	157	81.8	298	91.4	118	79.7

Reimbursement	2	0.7	17	6.7	7	2.0	6	3.1	6	1.8	13	8.8

Health insurance	0	0	6	2.4	1	0.3	3	1.6	5	1.5	10	6.8

Installment	1	0.4	19	7.5	23	6.6	17	8.9	14	4.3	1	0.7

In-kind	0	0	1	0.4	3	0.9	0	0	0	0	1	0.7

Others	0	0	0	0	0	0	0	0	0	0	0	0

Table 5 shows the different payment-coping mechanisms that the respondents used to pay for health care. The use of own money was the commonest payment-coping mechanism in the three communities. However, a sizeable proportion of the respondents in three communities (Ezilo, Nkalagu and Udi) borrowed money in order to pay for care. The sale of movable household assets or land was not commonly employed while fee exemptions and subsidies were almost non-existent as coping mechanisms. In case of other household members, households mostly used their own money to pay for the healthcare services, although there was some appreciable use of borrowed money to pay for healthcare in some rural areas (Table 5).

**Table 5:** Payment coping mechanisms

Payment coping mechanisms for respondents												
	**Abakaliki (urban)**		**Ezilo (semi-urban)**		**Nkalagu (rural)**		**Eke-na-ene (rural)**		**Enugu (urban)**		**Udi (semi-urban)**	
	**N = 229**		**N = 244**		**N = 333**		**N = 209**		**N = 321**		**N = 174**	
	**n**	**(%)**	**n**	**(%)**	**n**	**(%)**	**n**	**(%)**	**n**	**(%)**	**n**	**(%)**

Own money	210	91.7	181	74.1	283	85.0	190	90.9	311	96.9	144	82.8

Borrowed money	3	1.3	34	13.9	38	11.4	4	1.9	2	0.6	44	25.3

Sold households' assets	1	0.4	20	8.2	1	0.3	1	0.5	0	0	4	2.3

Sold land	0	0	0	0	1	0.3	0	0	0	0	1	0.6

Someone else paid	11	4.8	4	1.6	7	2.1	7	3.3	8	2.5	8	4.7

Was exempted from payment	3	1.3	1	0.4	1	0.3	0	0	2	0.6	2	1.2

Payment was subsidised	1	0.4	2	0.8	2	0.6	0	0	1	0.3	0	0

Others	0	0	2	0.8	9	2.4	7	3.3	3	0.9	1	0.6

**Payment coping for other household members**												

	**N = 277**		**N = 273**		**N = 348**		**N = 160**		**N = 312**		**N = 179**	
	**n**	**(%)**	**n**	**(%)**	**n**	**(%)**	**n**	**(%)**	**n**	**(%)**	**n**	**(%)**

Own money	268	96.8	192	70.3	300	86.2	150	93.8	302	96.8	125	69.8

Borrowed money	8	2.8	27	9.9	36	10.3	6	3.8	2	0.6	42	23.5

Sold households' assets	1	0.4	41	15.0	2	0.6	0	0	0	0	3	1.7

Sold land	0	0	0	0	1	0.3	1	0.6	0	0	1	0.6

Community solidarity	0	0	2	0.7	5	1.4	0	0	0	0	1	0.6

Was exempted from payment	0	0	2	0.7	0	0	1	0.6	3	1.0	1	0.6

Payment was subsidised	0	0	5	1.8	3	0.9	0	0	2	0.6	2	1.1

Others	0	0	4	1.5	1	0.3	2	1.2	3	1.0	4	2.2

### Socio-economic status differentials in use of OOPS and in coping strategies

In most cases, respondents belonging to different SES groups used OOPS equally to pay for healthcare with the exception of Abakiliki where the most poor had the lowest proportion of people that used the mechanism (Table 6). However, for other household members there was a uniform statistically significant SES difference in the use of OOPS to pay for healthcare in the six study areas. The most-poor group was less likely to use OOPS as a payment mechanism compared to better-off SES groups. Based on the pooled data from the six sites, although OOPS was the most highly used payment mechanism by all SES groups, the most poor used reimbursement (p = 0.001), in-kind (p = 0.035) and installment (p = 0.06) more than other SES groups. Both the inter-quartile ratios and the concentration indices support the trend of inequity in use of OOPS to pay for healthcare. The results also show that as SES increases, households use more of own money to pay for healthcare. Also, as SES quartile decreased, the households sold their assets to pay for healthcare in Ezilo. The better-off quartiles were more able to borrow to pay for healthcare in Ezilo and Nkalagu, while the converse was true in Eke-na-ene.

**Table 6:** SES differences in use of OOPS for respondents and other household members

	Respondents n (%)	Other household members n (%)
Abakiliki	N (%)	N (%)
Q1 (most poor)	47 (22)	52 (20)
Q2 (very poor)	60 (29)	61 (22)
Q3 (poor)	45 (21)	76 (29)
Q4 (least poor)	58 (28)	76 (29)
Chi-square	7.16*	24.21***
Poor-rich ratio	0.81	0.68
Concentration index	0.03	0.10

Ezilo		
Q1 (most poor)	46 (22)	42 (20)
Q2 (very poor)	48 (23)	48 (24)
Q3 (poor)	59 (29)	57 (28)
Q4 (least poor)	52 (26)	57 (28)
Chi-square	4.25	7.32*
Poor-rich ratio	0.88	0.74
Concentration index	0.04	0.06

Nkalagu		
Q1 (most poor)	65(24)	63 (20)
Q2 (very poor)	61 (22)	76 (25)
Q3 (poor)	73 (27)	83 (27)
Q4 (least poor)	74 (27)	86 (28)
Chi-square	6.35*	22.55***
Poor-rich ratio	0.88	0.73
Concentration index	0.04	0.06

Eke-na-ene		
Q1 (most poor)	43 (23)	33 (21)
Q2 (very poor)	45 (25)	33 (21)
Q3 (poor)	51 (27)	47 (30)
Q4 (least poor)	45 (25)	44 (28)
Chi-square	2.35	9.38
Poor-rich ratio	0.50	0.03**
Concentration index	0.01	0.03

Enugu		
Q1 (most poor)	74 (24)	64 (22)
Q2 (very poor)	80 (26)	74 (25)
Q3 (poor)	78 (25)	76 (25)
Q4 (least poor)	78 (25)	83 (28)
Chi-square	2.66	16.14***
Poor-rich ratio	0.9	0.77
Concentration index	0.01	0.05

Udi		
Q1 (most poor)	32 (28)	24 (20)
Q2 (very poor)	26 (23)	22 (19)
Q3 (poor)	30 (27)	34 (29)
Q4 (least poor)	25 (22)	38 (32)
Chi-square	0.90	10.00**
Poor-rich ratio	1.28	0.63
Concentration index	-0.02	0.13

Note: * = p < 0.10; ** = p < 0.05; and ***p < 0.01

Logistic regression analysis showed that there were some statistically significant determinants of choice of OOPS for the payment of healthcare (Table 7). All the regression analyses were statistically significant. The expenditure on treatment was positively and statistically significantly correlated with use of OOPS in four of the communities, although the magnitudes of the coefficients were small. Females were less likely than men to use OOPS, although the finding was only statistically significant in Udi. Factors that had positive influence on use of OOPS were respondents being household heads in Udi, higher number of household residents in Eke-na-ene and Ezilo, years of schooling in Ezilo and transportation costs in Abakiliki and Eke-na-ene. However, transportation costs had a negative influence on use of OOPS in Ezilo. The weights of the SES indices were insignificant explanatory factors, with the exception of Ezilo, where it was negatively related to use of OOPS.

**Table 7:** Logistic regression analysis of out-of-pocket user fees versus independent variables

	Abakaliki Coeff (SE)	Ezilo Coeff (SE)	Nkalagu Coeff (SE)	Eke-na-ene Coeff (SE)	Enugu Coeff (SE)	Udi Coeff (SE)
Status in household	-.11 (.58)	-.37 (.58)	-.37 (.49)	-.80 (.63)	-.67 (.52)	.95 (.38)**

No of household residents	-.09 (.07)	.07 (.04)*	-.02 (.03)	.11 (.05)**	-.01 (.08)	.07 (.06)

Sex	-.35 (.58)	.18 (.51)	-.05 (.49)	.80 (.61)	-.22 (.53)	-.91 (.32)***

Age	.03 (.01)**	.003 (.01)	-.01 (.01)	-.0002 (.01)	.01 (.02)	-.02 (.01)

Years of schooling	.001 (.04)	.04 (.03)*	.02 (.04)	-.01 (.03)	.01 (.04)	-.04 (.04)

Marital status	-1.06 (.66)	-1.2 (.69)*	.59 (.70)	1.00 (.87)	.43 (.62)	1.30 (1.16)

Cost of treatment	.01 (.001)***	.001 (.0002)***	.0004 (.0002)**	.0002 (.0002)	.0002 (.0001)	.0004 (.0002)*

Transport costs	.02 (.006)***	.002 (.001)	-.002 (.001)*	.02 (.004)***	-.002 (.001)	-.001 (.002)

SES index	.04 (.10)	-.18 (.10)*	.09 (.10)	.05 (.10)	.09 (.15)	.04 (.10)

Constant	-.56 (.88)	.24 (.99)	.94 (.83)	-1.43 (1.01)	1.26 (.96)	-1.38 (1.31)

LR chi2	197.6***	64.4***	20.0**	40.7***	11.46***	22.31***

Pseudo R2	0.39	0.13	0.05	0.11	0.05	0.06

No of correct predictions	86.52%	67.21%	73.40%	69.80%	88.64%	65.86%

Significance of parameters * <0.10, **<0.05, ***<0.01

## Discussion

The results indicate that most respondents of the study communities used OOPS as the commonest type of payment mechanism for health care consumption. However, this could be due to absence of wide-scale payment alternatives to OOPS. It is possible that if NHIS and other pre-payment (especially health insurance) mechanisms were widely available, payment by OOPS would not be so high. The limited use of payment mechanisms such as reimbursement, installment and in-kind payments either reflect their low acceptability by providers or a low level of awareness that they could be used by the consumers.

In the study area, OOPS appeared largely uninfluenced by socio-economic status (SES). Hence, the differences in relative use of different providers had no relationship with use of OOPS. However, OOPS was found to be more significantly used by the urban communities while the rural communities used more of reimbursement, installment payment and in-kind payment mechanisms. This may be because the urban dwellers are more educated and have more assets and economic power. The lack of SES differentials in use of OOPS by respondents implies poor people are suffering and are not protected from the hazards and uncertainty of paying for healthcare when ill. This can lead to individuals to either delay or not seek healthcare at all. Some of the inequity might be explained by urban-rural differences especially taking into consideration that the most-poor people usually reside in the rural areas and options for healthcare payments are also more limited in rural areas.

User fees paid through OOPS, which has been universally recognised to be very retrogressive, was the most common payment mechanism used to pay for care by all the SES quartiles in different geographic settings. The impact of OOPS is worse on the poorest households as they are more likely to have higher occurrences of catastrophe due to health payments through OOPS. Payment mechanisms that engender equity should be developed and made part and parcel of the healthcare system. Countries with more developed economies have managed to move away from user fees for health care towards social insurance or tax-based models [26]. Although the NHIS has enrolled the federal government civil servants, the NHIS was rarely used by the study communities indicating that the mechanism is still rare in the country. The irony is that the countries whose populations can least afford to pay for health care are the ones who still rely on user fees even though it is a sub-optimal method of financing. However, experiences in other countries show that user fees can be abolished successfully [11,26,17]. After all, the income that they are bringing in is not is usually not substantial. Nonetheless, the principal questions with regards to elimination of user fees are still: how to appropriately fill the financing gap resulting from such elimination, how to provide evidence that abolition is responsible for better access to modern healthcare; and to determine if there are other important reasons that might have negative impact on the demand for modern healthcare (e.g. low quality, bribes).

A sizeable proportion of the respondents in the three rural communities in the present study borrowed money in order to pay for health care. Borrowing to pay medical fees has been reported in a number of studies [27]. The adoption of specific short-term responses to crisis events such as sale of land or movable household assets and taking a loan were not commonly used to cope with payment for healthcare in the present study. It seems that households therefore make decisions which reflect trade-offs between competing objectives. Research on households' responses to drought and famine suggests that households often adopt coping strategies which aim to protect the viability of future livelihoods, demonstrating an "awareness of a future beyond the current crisis when assets may be needed for other purposes" [28]. Lands are often considered as 'core' assets, which are essential to maintaining current and future incomes, as opposed to 'surplus' assets which are primarily a store of value. Disposal of 'surplus' assets does not lead households to incur catastrophic opportunity costs through a decline in subsequent incomes, and is more easily reversible. However, disposal of 'core' productive assets may lead to a 'poverty ratchet' [29,30] in which a household is unable to protect itself from subsequent financial shocks.

It was not surprising that taking a loan was not a popular coping mechanism adopted by the respondents. The reason may be that formal credit institutions employ screening devices to overcome information and incentive problems [31], and this often results in the exclusion of poor households from access to formal credit. Most poor households may not qualify for loans as loans are usually made available to those with the ability to repay as assessed by a credit officer. Many households with the capacity to obtain loans may be unwilling to seek formal loans to pay medical bills due to high interest rate and the fear that they may find it difficult to repay the loans in the future. Similarly, community solidarity was not a popular coping mechanism even though it has been reported that it can overcome information asymmetries and incentive problems and as such can be used as informal credit market to households who are excluded from formal credit institutions [32,33]. There was very little evidence of actual existence of fee exemptions and other discretionary payment strategies that could be used to pay for the poorest and hence improve their level of access and utilization to healthcare services.

Removing user fees and limiting the use of OOPS for essential healthcare conditions and for control of endemic diseases should be regarded as part of a package to improve quality and sustainability of health services. These should be complemented by implementing appropriate health insurance schemes to cover the informal sector and ensure financial risk protection of the poor in the context the National Health Insurance Scheme. The abolition of user fees is also cited by the Millennium Project report as a necessary prerequisite for achieving the health-related Millennium Development Goals (MDGS) [34]. However, as some authors cautioned from the experiences in Uganda, the removal of user fees should be accompanied by drug availability and improved quality of services [11]. It is however noteworthy that not all health systems will be able to remove user fees immediately, nor will removal of fees be successful without complementary supply-side reforms.

In the right place and manner, abolition of user fees through OOPS can be part of a powerful vicious circle as it can improve access and outcomes. Studies in Burkina Faso [35] and elsewhere [36-38] have shown that the introduction of fees resulted in deterring people, especially the poor and children, from using health care services. In contrast to this, a widely cited study from Cameroon found that the poor used more state-provided health care despite fee increases as a result of quality improvements [39]. Similarly, growing demand after introduction of cost recovery and better quality of care was found in a pilot study in Niger [40].

## Conclusion

This study has demonstrated the existence of SES inequities in use of OOPS to pay for healthcare services. Developing equitable financing approaches will depend on the assessment of the burden and determinants of OOPS on healthcare seeking by different socio-economic and geographic groups, leading to determining how best to protect the poor. Innovative approaches that would reduce the inequity in financial access to and utilization of healthcare services by the poor are needed, especially if the health-related millennium development goals (MDGs) are to be met in Nigeria. However, the attainment of the MDGs in health could be hampered by OOPS.

Equity can be achieved if public expenditures are targeted to poorer and underserved sections of the society, and if an appropriate financial risk protection mechanism(s) are adopted across the states and the country in general, especially within the context of the National Health Insurance Scheme. In order to avoid the negative incentives of OOPS on those who are unable (or unwilling) to pay, user fee exemptions can provide a way of avoiding the negative equity impacts of such a policy [2]. Hence, discretional fees and quality improvements of services at reduced costs are additional strategies for enhancing financial equity in access and utilization of healthcare services.

The body of knowledge on how OOPS affect equitable access to care and utilization of services in Nigeria and in many sub-Saharan African countries needs to be continually increased so as to help improve evidence-based decision making and accelerate efforts of policy makers to move away from OOPS to more insurance-based payment mechanisms. In addition, this will help ensure that people do not have to pay out-of-pocket at point of consumption of healthcare services. Future studies should examine other dimensions of incidence (such as geographic differences) and consequences of OOPS such as catastrophe and impoverishment in different settings amongst different population groups. Future studies that would examine the determinants of OOPS should also broaden the inquiry to include alternatives such as people refraining from seeking healthcare.

## Competing interests

The authors declare that they have no competing interests.

## Authors' contributions

OO conceived the study and wrote the first draft of the paper. All the co-authors participated in data collection, data analysis and interpretation of the data, as well as critical revision of the drafts of the paper. All authors read and approved the final manuscript.

## Pre-publication history

The pre-publication history for this paper can be accessed here:

http://www.biomedcentral.com/1472-6963/10/67/prepub

## Supplementary Material

Additional file 1 Household Questionnaire. Click here for file
