# Supplementary material for: Investigating determinants of out-of-pocket spending and strategies for coping with payments for healthcare in southeast Nigeria
Source: BMC Health Serv Res. 2010 Mar 17;10:67. doi: 10.1186/1472-6963-10-67 (PMC2851710; doi:10.1186/1472-6963-10-67)
Supplement: Additional file 1 — Household Questionnaire. [file 1472-6963-10-67-S1.DOC]

### **Household Questionnaire**

Dear respondent,

We are interested in your opinion about the diseases that trouble this area and how to control them. To achieve this aim, we wish to conduct a questionnaire survey on your experiences in preventing and treating them. You shall in the future get the results of this exercise, which we hope to use to help this area and the government to control the diseases and improve your health. All information given will be private. Your participation is voluntary, and you do not have to answer questions you do not want to answer.

***Instruction: Please fill the next section at the beginning and at the end of the interview.***

What is the respondent's home address_______________________

What is the name of the interviewer When was the date of interview < / / >

What time did the interview start: _____________: What time did the interview end:________________

**Please, enter the appropriate number representing the answer given in the spaces provided.**

### **1: DEMOGRAPHIC INFORMATION:**

### **I would like to begin by asking a few questions about you and your household.**

1. What is your status in this household? [ ] 1 = household head. 0 = representative of household

*[Note:* ***Only*** *the spouse, or the head of household in her absence should be interviewed.]*

2. How many people live in this household, including yourself? [ ]

3. How many adults (greater than or equal to 18 years) live here? _______No. of adults

4. How many teenagers (12-17 years) live here? _______ No. of teenagers

5. How many children (5-11 years) live here? _______ No. of children

6. How many very small children (less than 5 years) live here? _______ No. of small children

*6a. Enumerator: Add the responses to questions 3,4,5 and 6 and check that the sum is the same as the response to question 2)* ______ Total of questions 3,4,5, and 6

8. What is your name? ______________________________

9. [Enumerator: What is the gender of the respondent?] [ ] 1 = male 0 = female

10. How old are you? [ ]

11. Did you go to school? [ ] 1 = yes 0 = no

12. If #11 is yes, what was the total number of years that you spent schooling? [ ]

13. What occupation is your major source of getting money? [ ]

14. Are you married [ ] 1 = yes 0 = no

15. What is your current marital status? [ ] 1 = Single. 2 = married. 3 = widowed. 4 = divorced/separated.

**2a. Diseases prioritization**

16.What do you think is the most important disease or health condition that people visit health facilities for in this area? *[Enumerator: Do not read list of responses. Record first response only. Mark only one.]* 1 = yes 0 = no

16a. Malaria [ ]

16b. Tuberculosis [ ]

16c. Respiratory problems, not including tuberculosis (for example, asthma or bronchitis) [ ]

16d. Diarrhea [ ]

16e. Hypertension [ ]

16f. Appendicitis/ hernia [ ]

16g. Malnutrition [ ]

16h. HIV/AIDS [ ]

16i. Antenatal care [ ]

16j. Child-birth [ ]

16k. Other (please specify) __________________ [ ]

**2b. Health seeking**

18. Which diseases or health conditions did you and or other member(s) of your household have in the past month?

*[Enumerator: Do not read list of responses. Mark as many as mentioned 1 = yes 0 = no]*

18a. Malaria [ ]

18b. Tuberculosis [ ]

18c. Respiratory problems, not including tuberculosis (for example, asthma or bronchitis) [ ]

18d. Diarrhea [ ]

18e. Hypertension [ ]

18f. Appendicitis/ hernia [ ]

18g. Malnutrition [ ]

18h. HIV/AIDS [ ]

18i. Antenatal care [ ]

18j. Child-birth [ ]

18k. Other (please specify) __________________ [ ]

19. How did you know that either you or somebody in your household was sick? 1 = yes 0 = no *(Enumerator: Mark first response only.)*

19a. Laboratory tests [ ]

19b.Community health worker told me [ ]

19c.Traditional healer told me [ ]

19d.I recognized the symptoms myself [ ]

19e. A family member told me that I was sick [ ]

19f. Others [ ] Please specify _______________________________________

19g.Don’t know / Don’t remember[ ]

20. How many times were you or any member of your household sick over the past 6 months?

20a. No. of times for respondent [ ]

20b. No. of times for other adults [ ]

20c. No of times for teenagers [ ]

20d. No of times for children [ ]

20e. No of times for members of the household [ ] *(Enumerator: Add responses to questions 20a to20e; If zero, go to 53]*

**2c. Health seeking and cost of illness (respondent)** I want to talk with you about the most recent episode (within the past month).

21. When was the last time you were sick or had a health condition that needed treatment or care in the past one month? Date: -----/------/--------

22. What was the type of sickness or health condition? ______________________

23. How many days were you sick? [ ] days

24. Did you seek treatment?[ ] 1 = yes 0 = no(GO TO 46)

25.What treatment did you first seek? 1 = yes 0 = no *[Enumerator: Do not read list. Mark first response only.] (Write down the name and address of the provider(s) visited)*

25a. Traditional medicines: [ ] _____________________________________________________________

25b. Went to chemist (patent medicine dealer): [ ]______________________________________________

25c. Went to the Community health worker:[ ] _________________________________________________

25d. Went to the health center: [ ]_________________________________________________________

25e. Went to the hospital or clinic: [ ] ________________________________________________________

25f. Clean the environment:[ ] ___________________________________________________________

25g. Other: [ ] (specify:) ___________________________________________________________________

26. Why did you seek treatment where you did? *[Enumerator: Multiple answers are allowed]* 1 = yes 0 = no

26a. Good services provided [ ]

26b. Readily available drugs [ ]

26c. Near [ ]

26d. Affordable services [ ]

26e. Prompt attention [ ]

26f. Polite health workers [ ]

26g. Others (specify) [ ] ______________________________________________________

28. How much did it cost to receive this treatment not including the cost of transportation? ___________Naira

29. How much did you spend on transportation? _________________ Naira

30. Total cost [*Enumerator add 28 + 29]* _________________ Naira

31. How was the treatment cost paid? I will read out some payment options and please answer either yes or no to each option. 1 = yes 0 = no *[Enumerator: Multiple responses are allowed]*

31a. Paid (cash and carry) but was reimbursed by employer [ ]

31b. Cash and carry [ ]

31c. Private health Insurance [ ]

31d. National Health Insurance [ ]

31e. Installment [ ]

31f. In-kind [ ] Please specify ______________________________________

31g. Pre-payment [ ]

31h. Others (please specify) [ ] _______________________________________

32. How did you cope with the payment? I will read out some options and answer either yes or no to each option. *(Enumerator: Multiple responses are allowed)*Coding of payment coping mechanisms 1 = yes 0 = No

32a. Own money [ ]

32b. Borrowed money [ ]

32a. Sold household movable assets [ ]

32d. Sold family land [ ]

32e. Took a loan [ ]

32f. Community solidarity [ ]

32g. Someone else paid [ ]

32h. Was exempted from payment [ ]

32i. Payment was subsidised [ ]

32j. Others (specify) ___________________________________

33. How did your household cope with your sickness, i.e. the functions that you usually performed? I will read out some options and please answer either yes or no to each option. *(Enumerator: Multiple responses are allowed)*

Coding of illness coping mechanisms [ 1 = yes 0 = No]

33a. Intra-household labour substitution [ ]

33b. Hired someone to do the work [ ]

33c. Ill person continued to perform his normal functions [ ]

33d. Others (specify) [ ]

34. Did you recover? [ ] 1 = yes (go to 46) 0 = no

35. If you did not recover, what action did you take next? *(Enumerator :Do not read list. Mark first response only. Write down the name of the provider visited)*

35a. Traditional medicines: [ ] _____________________________________________________________

35b. Went to the clinic: [ ] _______________________________________________________________

35c. Went to chemist (patent medicine dealer): [ ]_____________________________________________

35d. Went to the Community health worker:[ ] ________________________________________________

35e. Went to the health center: [ ]__________________________________________________________

35f. Went to the hospital: [ ]______________________________________________________________

35g. Clean the environment:[ ] __________________________________________________________

35h. Other: [ ] (specify:) __________________________________________________________________

36. In the second action you took, how much did it cost to receive this treatment not including the cost of transportation? _________ Naira

37. How much did you spend on transportation? _________________ Naira

38. Total cost [*Enumerator add 36 + 37]* _________________ Naira

39. How was the treatment cost paid? I will read out some payment options and please answer either yes or no to each option. 1 = yes 0 = no *[Enumerator: Multiple responses are allowed]*

39a. Paid (cash and carry) but was reimbursed by employer [ ]

39b. Cash and carry [ ]

39c. Private health Insurance [ ]

39d. National Health Insurance [ ]

39e. Installment [ ]

39f. In-kind [ ] Please specify ______________________________________

39g. Pre-payment [ ]

39h. Others (please specify) [ ] _______________________________________

40. How did you cope with the payment? I will read out some options and answer either yes or no to each option. *(Enumerator: Multiple responses are allowed)*Coding of payment coping mechanisms 1 = yes 0 = No

40a. Own money [ ]

40b. Borrowed money [ ]

40a. Sold household movable assets [ ]

40d. Sold family land [ ]

40e. Took a loan [ ]

40f. Community solidarity [ ]

40g. Someone else paid [ ]

40h. Was exempted from payment [ ]

40i. Payment was subsidised [ ]

40j. Others (specify) ___________________________________

41. How did your household cope with your sickness, i.e. the functions that you usually performed? *(Enumerator: Multiple responses are allowed) C*oding of illness coping mechanisms [ 1 = yes 0 = No]

41a. Intra-household labour substitution [ ]

41b. Hired someone to do the work [ ]

41c. Ill person continued to perform his normal functions [ ]

41d. Others (specify) [ ]

42. If you did not recover, what action did you take next? *(Enumerator: Write down the name of the provider(s) visited and actions taken until the patient finally recovered)*

43. What was the total cost of treatment (transportation/drugs/others) until you recovered? **___________**

*44. Table summarizing households’ expenditure to treat respondents’ illness in last month.* (Enumerator: Complete the table using all the information provided above)

|  | Where visited | Diagnosis given | Transportation cost | Treatment cost | Total cost |
| --- | --- | --- | --- | --- | --- |
| 1^st^ action |  |  |  |  |  |
| 2^nd^ action |  |  |  |  |  |
| 3^rd^ action |  |  |  |  |  |
| Other actions until he/she recovered |  |  |  |  |  |
| Total |  |  |  |  |  |

*45. Table summarizing households' coping mechanisms for fee payment and the normal functions of the respondents' illness in last month.* (Enumerator: Complete the table using all the information provided above)

|  | Payment coping | Functions coping |
| --- | --- | --- |
| 1^st^ action |  |  |
| 2^nd^ action |  |  |
| 3^rd^ action |  |  |
| Other actions until s/he recovered |  |  |

**2d. Health seeking and cost of illness (other household members)**

Now I'd like to ask you about other household members beside yourself and their experience with sickness.

46. In total, how many *other household members were* sick over the past 6 months? ______ No. of household members (*Enumerator: If nobody was ill or had a health condition, go to Section 3and ask Q53).*

47. Which diseases did they have in the past month?

*[Enumerator: Do not read list of responses. Mark as many as mentioned]*

47a. Malaria [ ]

47b. Tuberculosis [ ]

47c. Respiratory problems, not including tuberculosis (for example, asthma or bronchitis) [ ]

47d. Diarrhea [ ]

47e. Hypertension [ ]

47f. Appendicitis/ hernia [ ]

47g. Malnutrition [ ]

47h. HIV/AIDS [ ]

47i. Antenatal care [ ]

47j. Child-birth [ ]

47k. Other (please specify) __________________ [ ]

48. What actions were taken for each person that was sick and how much was spent?

|  | Age (years) | Diagnosis given | Transport cost | Treatment cost | Transport + treatment cost |
| --- | --- | --- | --- | --- | --- |
| 48a. Name (case1):  48a1. Type of illness  48a2. 1^st^ action:  48a3. 2^nd^ action:  48a4 Other actions (until he/she recovered):  48a5. Total |  |  |  |  |  |
| 48b. Name (case2):  48b1. Type of illness  48b2. 1^st^ action:  48b3. 2^nd^ action:  48b4. Other actions (until he/she recovered):  48b5. Total |  |  |  |  |  |
| 48c. Name (case3):  48c1. Type of illness:  48c2. 1^st^ action:  48c3. 2^nd^ action:  48c4. Other actions (until he/she recovered):  48c5. Total |  |  |  |  |  |
| 48d. Name (case4):  48d1. Type of illness  48d2. 1^st^ action:  48d3. 2^nd^ action:  48d4. Other actions (until he/she recovered):  48d5. Total |  |  |  |  |  |
| 48e.Total cost |  |  |  |  |  |

49. How was the treatment cost paid? I will read out some options and please answer either yes or no to each option. = yes 0 = no *[Enumerator: Multiple responses are allowed]*

49a. Paid (cash and carry) but was reimbursed by employer [ ]

49b. Cash and carry [ ]

49c. Private health Insurance [ ]

49d. National Health Insurance [ ]

49e. Installment [ ]

49f. In-kind [ ] Please specify ______________________________________

49g. Pre-payment [ ]

49h. Others (please specify) [ ] _______________________________________

50. How did you cope with the payment? I will read out some payment options and please answer either yes or no to each option. *(Enumerator: Multiple responses are allowed)* Coding of payment coping mechanisms [ 1 = yes 0 = No]

50a. Own money [ ]

50b. Borrowed money [ ]

50a. Sold household movable assets [ ]

50d. Sold family land [ ]

50e. Took a loan [ ]

50f. Community solidarity [ ]

50g. Someone else paid [ ]

50h. Was exempted from payment [ ]

50i. Payment was subsidised [ ]

50j. Others (specify) ___________________________________

51. How did your household cope with the sickness of the first person, i.e. the functions that s/he usually performed? *(Enumerator: Multiple responses are allowed) C*oding of illness coping mechanisms 1=yes 0=No

51a. Intra-household labour substitution [ ]

51b. Hired someone to do the work [ ]

51c. Ill person continued to perform his normal functions [ ]

51d. Others (specify) [ ]

*52. Table summarizing households' coping mechanisms for fee payment and the normal functions of other household members in last month.* (Enumerator: Complete the table using all the information provided above)

|  | Payment coping | Function coping |
| --- | --- | --- |
| 52a. Name (youngest; Case:1):  52a1. Type of illness  52a2. 1^st^ action:  52a3. 2^nd^ action:  52a4. Other actions (until he/she recovered):  52a5. Total |  |  |
| 52b. Name (case2):  52b1. Type of illness  52b2. 1^st^ action:  52b3. 2^nd^ action:  52b4. Other actions (until he/she recovered):  52b5. Total |  |  |
| 52c. Name (case3):  52c1. Type of illness  52c2. 1^st^ action:  52c3. 2^nd^ action:  52c4. Other actions (until he/she recovered):  52c5. Total |  |  |

**3. Fee exemptions**. Now, I would like to ask you about the knowledge and experiences that you and other household members have about fee exemptions.

53. Are you aware of the existence of fee exemptions for certain people and diseases in health facilities? [ ] 1 = yes (go to Q54) 0 = no (go to Q69)

54. Which health facilities do you know that give fee exemptions? *(Enumerator: Write down the name and address of the provider(s) mentioned)* 1 = yes 0 = no

[ ] (54a) Traditional medicine healers: ______________________________________________________

[ ] (54b) Clinic: _________________________________________________________________________

[ ] (54c) Chemist (patent medicine dealer): ____________________________________________________

[ ] (54d) Community health worker: _________________________________________________________

[ ] (54e) Health center: ____________________________________________________________________

[ ] (54f) Hospital: _______________________________________________________________________

[ ] (54g) Other (specify:) ___________________________________________________________________

55. What type of goods and services do they exempt? I will read out some options and please answer either yes or no to each option. 1 = yes 0 = no

55a. Registration fees [ ]

55b. Consultation fees [ ]

55c. Laboratory fees [ ]

55d. Drug fees [ ]

55e. Bed fees (for in-patients) [ ]

55f. Preventive services [ ]

55g. Others (specify) [ ] __________________________________________________________________

56. What categories of people are exempted from paying the fees? 1 = yes 0 = no

56a. Children [ ]

56b. Adults [ ]

56c. Poor people [ ]

56d. Widows/widowers [ ]

56e. Pregnant women [ ]

56f. Others (specify) [ ] _________________________________________________________________

57. What diseases or conditions are exempted? 1 = yes 0 = no

57a. Malaria [ ]

57b. Tuberculosis [ ]

57c. Respiratory problems, not including tuberculosis (for example, asthma or bronchitis) [ ]

57d. Diarrhea [ ]

57e. Hypertension [ ]

57f. Appendicitis/ hernia [ ]

57g. Malnutrition [ ]

57h. HIV/AIDS [ ]

57i. Antenatal care [ ]

57j. Child-birth [ ]

57k. Other (please specify) __________________ [ ]

58. Have you ever been exempted from fees? [ ] 1 = yes 0 = no (Go to Q64)

59. Why were you exempted from fees? *(Enumerator: Write down the reasons given)*

_____________________________________________________

60. Where were you exempted from fees? 1 = yes 0 = no (*Enumerator: Multiple answers are allowed. Add address of provider)*

[ ] (61a) Traditional medicine healers: ______________________________________________________

[ ] (61b) Clinic: _________________________________________________________________________

[ ] (61c) Chemist (patent medicine dealer): ____________________________________________________

[ ] (61d) Community health worker: _________________________________________________________

[ ] (61e) Health center: ____________________________________________________________________

[ ] (61f) Hospital: _______________________________________________________________________

[ ] (61g) Other (specify:) ___________________________________________________________________

62. What type of goods and services was/were exempted? 1 = yes 0 = no

62a. Registration fees [ ]

62b. Consultation fees [ ]

62c. Laboratory fees [ ]

62d. Drug fees [ ]

62e. Bed fees (for in-patients) [ ]

62f. Others (specify) [ ] __________________________________________________________________

63. What disease(s) or condition did you have then that was/were exempted? 1 = yes 0 = no

63a. Malaria [ ]

63b. Tuberculosis [ ]

63c. Respiratory problems, not including tuberculosis (for example, asthma or bronchitis) [ ]

63d. Diarrhea [ ]

63e. Hypertension [ ]

63f. Appendicitis/ hernia [ ]

63g. Malnutrition [ ]

63h. HIV/AIDS [ ]

63i. Antenatal care [ ]

63j. Child-birth [ ]

63k. Other (please specify) __________________ [ ]

64. How many of your other household members have ever been exempted from fees? [ ] (*Enumerator: if zero, go to Q69)*

65. Why were they exempted from fees? *(Enumerator: Write down the reasons given)* _____________________________________________________

66. Where were they exempted from fees? 1 = yes 0 = no (*Enumerator: Multiple answers are allowed)*

[ ] (66a) Traditional medicine healers: ______________________________________________________

[ ] (66b) Clinic: _________________________________________________________________________

[ ] (66c) Chemist (patent medicine dealer): ____________________________________________________

[ ] (66d) Community health worker: _________________________________________________________

[ ] (66e) Health center: ____________________________________________________________________

[ ] (66f) Hospital: _______________________________________________________________________

[ ] (66g) Other (specify:) ___________________________________________________________________

67. What type of goods and services was/were exempted? 1 = yes 0 = no

67a. Registration fees [ ]

67b. Consultation fees [ ]

67c. Laboratory fees [ ]

67d. Drug fees [ ]

67e. Bed fees (for in-patients) [ ]

67f. Others (specify) [ ] __________________________________________________________________

68. What disease(s) or conditions did they have then that was/were exempted? 1 = yes 0 = no

68a. Malaria [ ]

68b. Tuberculosis [ ]

68c. Respiratory problems, not including tuberculosis (for example, asthma or bronchitis) [ ]

68d. Diarrhea [ ]

68e. Hypertension [ ]

68f. Appendicitis/ hernia [ ]

68g. Malnutrition [ ]

68h. HIV/AIDS [ ]

68i. Antenatal care [ ]

68j. Child-birth [ ]

68k. Other (please specify) __________________ [ ]

**4 WEEKLY FOOD EXPENDITURES** *Next I'd like to ask you about things you spend your money on each week.*

69. How much did your household spend to purchase food from the market in the past one week on the various items that I will read out?

| Item | Quantity | Who purchased | Amount |
| --- | --- | --- | --- |
| 69a. Gari  69b. Beans  69c. Cassava (akpu)  69d. Fio-Fio (Ngbugbu)  69e. Cocoyam (ede)  69f. Rice  69g. Corn  69h. Fish  69i. Meat  69j. Vegetables  69k. Others (specify)  69l. Total |  |  |  |

70. If the food items that your household produced, but also consumed in the past one week were bought from the market, how much will they cost?

| Item | Quantity | Amount | **Total** |
| --- | --- | --- | --- |
| 70a. Gari  70b. Beans  70c. Cassava (akpu)  70d. Fio-Fio  70e. Cocoyam (ede)  70f. Rice  70g. Corn  70h. Fish  70i. Meat  70j. Vegetables  70k.Others (specify)  70l. Total |  |  |  |

71. Total food cost (Enumerator: Add 69 + 70) [ ] Total food cost

**5. HOUSEHOLD ASSETS** Could you tell me if someone in your household owns a(n) *(category name)*? *(Enumerator: In each category, indicate whether the item is owned by the respondent or a household member)* 1 = yes 0 = no

**72. Electronics**

72a. Radio [ ]

72b. Fridge [ ]

72c. Television [ ]

**73 Transport**

73a. Bicycle [ ]

73b. Motorcycle [ ]

73c. Motorcar [ ]

**74. Model of house**

74a. Up-stair [ ]

74b. Bungalow [ ]

**75. Major source of lighting**

75a. Electricity [ ]

75b. Kerosene lamp [ ]

75c. Others (specify) [ ] _______________________________________

**Thank you**
